# Comparison of virulence and resistance genes in *Mannheimia haemolytica* and *Pasteurella multocida* from dairy cattle with and without bovine respiratory disease

**DOI:** 10.1128/spectrum.01200-25

**Published:** 2025-06-16

**Authors:** Adriana Garzon, Craig Miramontes, Bart C. Weimer, Rodrigo Profeta, Alejandro Hoyos-Jaramillo, Heather M. Fritz, Richard V. Pereira

**Affiliations:** 1 Department of Population Health and Reproduction, School of Veterinary Medicine, University of California 70733, Davis, California, USA; 2 100K Pathogen Genome Project, School of Veterinary Medicine, University of California 70733, Davis, California, USA; 3 California Animal Health and Food Safety Lab, University of California 8789, Davis, California, USA; Michigan State University, East Lansing, Michigan, USA

**Keywords:** dairy cattle, antimicrobial resistance, whole-genome sequencing, WGS, BRD

## Abstract

*Mannheimia haemolytica* and *Pasteurella multocida* are two of the main bacterial pathogens associated with bovine respiratory disease (BRD). BRD represents one of the most significant health challenges in the cattle industry, causing substantial economic losses through animal morbidity and mortality while raising serious welfare concerns. The objectives of this project were to (i) characterize virulence factor (VF) and antimicrobial resistance (AMR) genes in *M. haemolytica* and *P. multocida* isolates from dairy cattle of different ages with and without BRD using whole-genome sequencing (WGS); (ii) evaluate associations between microbial genetic elements and animal disease status; and (iii) assess the accuracy of genome-based predictions for the antimicrobial resistance phenotype. Using a case-control study, AMR and VF genes were characterized from 149 *P*. *multocida* and 68 *M*. *haemolytica* isolates from preweaned calves, weaned heifers, and cows with and without BRD. The large genetic diversity observed in both bacterial species prevented the identification of unique genetic markers associated with disease status or age group. AMR genes (22 genes) from 12 antimicrobial classes were identified in *P. multocida* isolates, while 11 AMR genes for seven antimicrobial classes were identified in *M. haemolytica* isolates. Additionally, 28 and 15 virulence genes were identified in *P. multocida* and *M. haemolytica*, respectively. The ability of WGS-based predictions to predict phenotypic antimicrobial resistance showed variable accuracy across different antimicrobials, achieving moderate levels of agreement overall. Findings from this project demonstrate that identifying genomic markers based on gene presence/absence lacks discriminatory power within this population for identifying unique genotypes associated with disease status in these genomically diverse organisms.

**IMPORTANCE:**

This case-control study provides key microbial ecological advances by elucidating the role of bacteria in the bovine respiratory disease complex in dairy cattle. Previous research has identified specific virulence factors in both bacterial genomes that resulted in disease. Our results challenge this perception and are of high impact, revealing that the pan-genome of both bacteria did not differentiate among the clinical cases or age groups, and a specific pathogenic pathotype was not evident in the isolates from this study, and it did not emerge when including additional public whole-genome sequences to increase the analytical power of the analysis (the first study to use this approach to evaluate bovine respiratory disease in cattle). In addition to these novel discoveries, this study describes the first population-scale genomic comparison of both *Mannheimia haemolytica* and *Pasteurella multocida* genomes collected from affected and healthy dairy cattle from different age groups and from multiple farms.

## INTRODUCTION

Bovine respiratory disease (BRD) is a multicausal infectious disease and represents one of the most significant health challenges in the cattle industry, causing substantial economic losses through animal morbidity and mortality while raising serious welfare concerns. BRD is also an important driver of antimicrobial use on farms. Results from the 2014 USDA National Animal Health Monitoring Survey revealed that respiratory disorders affected 12% of preweaned calves, 5.1% of weaned heifers, and 2.8% of adult dairy cattle, with 94.8%, 91.8%, and 95% of these animals, respectively, receiving antimicrobial therapy for respiratory diseases (1). The cost of antimicrobial treatment for a preweaned calf diagnosed with BRD has been estimated to be $41.54 (2) per BRD case, with florfenicol and ceftiofur being the two drugs reportedly most used on California dairies (3). Therefore, research to improve the prevention and effective diagnosis and treatment of BRD in dairy cattle is an urgent need for the cattle industry.

*Mannheimia haemolytica* and *Pasteurella multocida* are bacterial pathogens associated with BRD. These two bacteria are gram-negative facultative anaerobes and commensal microorganisms of the upper respiratory tract of healthy cattle. Key virulence factors (VFs) of *M. haemolytica* and *P. multocida* include protein adhesins, capsular polysaccharides, outer membrane proteins, iron-binding proteins, lipopolysaccharides, enzymes, and leukotoxin, which work together to evade the host’s immune system, damage immune cells, and induce a strong inflammatory response (4, 5). Antimicrobial resistance (AMR) in both *M. haemolytica* and *P. multocida* isolated from BRD cases is a growing concern, especially with a trend for an increase in multidrug-resistant (MDR) isolates from sick animals (6–10).

Current genomic studies aiming to characterize *M. haemolytica* and *P. multocida* have mainly focused on the genes that underlie AMR and have used cross-sectional studies, with most of the studies using beef cattle populations. However, this approach leaves a gap in understanding AMR mechanisms, transmission, and prevalence in dairy cattle. A more comprehensive understanding of both VF and AMR profiles in these pathogens, particularly comparing isolates from BRD cases and healthy animals across different age groups, is essential for developing targeted therapeutic interventions and preventive measures.

The objectives of this study were to examine the genes related to virulence using whole-genome sequencing (WGS) and population comparative genomics that examined (i) the characterization of VF and AMR genes in *M. haemolytica* and *P. multocida* isolates from dairy cattle with and without BRD of different ages; (ii) the associations between these genetic elements and disease status; and (iii) the accuracy of genome-based predictions for antimicrobial resistance phenotypes.

## MATERIALS AND METHODS

### Study design

A case-control study was conducted on a convenience sample of three large (>1,000 lactating cows) commercial dairy farms in Northern California, where individual preweaned calves (up to 7 weeks of age), weaned heifers, and lactating cows with BRD were matched with control BRD-negative animals and sampled once using a deep nasopharyngeal swab approach (11).

### Case definitions

Once a month, a veterinarian visited each of the participating dairy herds and observed all preweaned calves, weaned heifers, and lactating cows from the hospital (cows that have received antimicrobial treatment with a milk withhold period). All BRD-suspect animals were further examined following a standardized procedure for each age group, which consisted of clinical assessment and BRD scoring, thoracic auscultation, lung ultrasound (only in preweaned calves), and collection of nasal swabs for culture and antimicrobial susceptibility testing. For the thoracic ultrasound in preweaned calves, an Easi-Scan:Go ultrasound with a linear probe and a wireless BUG OLED binocular headset viewer was used (IMV Imaging, Rochester, Minnesota).

Preweaned calves were considered BRD-suspect animals if they presented with BRD signs such as eye discharge, nasal discharge, cough, or abnormal respiration. A preweaned calf was confirmed as a BRD case when the California BRD cumulative score was ≥5 (12) and had abnormal findings at the thoracic auscultation, ultrasound, or both (13).

Weaned heifers were considered BRD-suspect animals if they presented with BRD signs such as nasal discharge, sunken eyes, cough, or abnormal respiration. A weaned heifer was confirmed as a BRD case when its cumulative score was ≥1, based on the validated scoring system for post-weaned dairy calves (14), and had abnormal findings at the thoracic auscultation (13).

Cows were considered BRD-suspect animals if they presented primary diagnostic criteria of BRD, such as cough, nasal discharge, and increased respiratory effort/rate, or secondary diagnostic criteria, such as depressed demeanor and low body condition score. A cow with at least two primary diagnostic criteria or one primary and one secondary was confirmed as a BRD case. Only females were included in the study. Animals were excluded if they were severely depressed, apathetic, or unable to stand and if they did not have any permanent identification or ear tag. Animal records regarding individual antimicrobial use were not available for the included animals.

After a case was identified, a control in the same herd matched by age and breed was selected randomly and further examined to confirm their BRD-negative status and enrolled as control. Animals were confirmed as a control if they showed normal findings on both clinical exam and thoracic auscultation. For preweaned calves, a lung ultrasound exam was also conducted to verify eligibility criteria.

### Sample collection

Samples were collected from both cases and controls in every age group. Samples were collected using a deep nasopharyngeal swab approach, as previously described (15). Briefly, samples were collected using a single-guarded sterile cotton swab (KI-3000, Kalayjian Industries, Inc., Signal Hill CA, USA) by restraining animals in a standing position, with the animals’ nostrils wiped clean with a single-use paper towel and subsequently disinfected with a 70% alcohol clean gauze before inserting the sterile swab medioventrally in the nasal cavity until nasopharyngeal tissue was reached and rotated several times against the mucosa. Swabs were immediately placed in Amies with charcoal transport media for bacterial isolation (ACM, BD BBL CultureSwab Plus Transport Systems, Franklin Lakes, NJ), and transported in a cooler with ice to the laboratory for processing (15).

### Bacterial isolation

Within 2 h after collection, samples were submitted to the California Animal Health & Food Safety Laboratory in Davis, California, for bacterial culture, speciation, and antimicrobial susceptibility testing. Each sample was cultured on sheep blood-3% agar (3% SBA) and chocolate agar and incubated for 48 h at 35 ± 2°C in 5% CO_2_. All colonies of interest were confirmed by biochemical testing and matrix-assisted laser desorption-ionization time-of-flight mass spectrometry, as previously described (16). Bacterial stocks were frozen at −80°C in glycerol until further analysis.

### DNA extraction, library preparation, and whole-genome sequencing

Previously frozen bacterial stocks were used to inoculate an SBA agar plate that was incubated aerobically at 37°C for 24 h. A single, well-isolated colony was subcultured in 10 mL of sterile brain heart infusion broth (Difco; Becton, Dickinson, and Company, Sparks, MD, USA) at 37°C for 24 h. Culture tubes were centrifuged at 10,000 × *g* for 3 min at room temperature (15°C–25°C), and the pellet was then resuspended in 180 µL nuclease-free water.

Total DNA was extracted as previously described (10, 17, 18) from the pellet using the Promega Wizard Genomic DNA (gDNA) Purification kit (Promega, Madison, WI, USA), following the manufacturer’s instructions for gram-negative bacteria, with slight modifications. All steps requiring centrifuging were done for 5 min. The DNA pellet was rehydrated with 100 µL of 10 mM Tris HCl solution overnight at 4°C. DNA concentration was measured at 260 nm using a NanoDrop One^c^ Microvolume UV-VIS spectrophotometer (Thermo Fisher Scientific, Inc., Waltham, MA).

DNA library preparation was conducted in Dr. Bart Weimer’s laboratory (UC Davis) as previously described (19–21). DNA was analyzed on the Agilent 2200 TapeStation System using the Genomic DNA ScreenTape assay for the integrity of gDNA. Libraries were constructed using the KAPA HyperPlus Library Preparation Kit (Roche, Indianapolis, IN, USA), as previously described (22, 23). Whole-genome sequencing was done using the Illumina HiSeq X platform with PE150 (Illumina Inc., San Diego, CA, USA) (24). All raw genome sequences generated in this study are available in the NCBI SRA under the BioProject accession number PRJNA186441.

### Sequence assembly, annotation, and pan-genome analyses

The genome sequences were assessed for sequencing depth (>20 × mean coverage), checked for quality using FastQC (v.0.11.9), and trimmed using Trimmomatic (v.0.39) (25). Sequences were assembled using Shovil (v.1.0.4) and checked for quality, genome size (2.0 to 2.8 Mbp), completeness (>95% estimate), and contamination (<5% estimate) using CheckM (26). Sequences were also assessed using Kraken2 with Braken to identify the genus and species (27). Four sequences did not meet the quality criteria and were removed from subsequent analysis.

The genomic comparative analyses were extended to include all available public genomes of *P. multocida* (*n* = 1,189) and *M. haemolytica* (*n* = 2,754). After the quality check, *P. multocida* (*n* = 1,082) and *M. haemolytica* (*n* = 2,753) genomes were used for genomic population comparisons. These genomes were combined with the genomes from our study to examine their genome-relatedness, following the same procedures described above.

The genomic similarities were computed using all-by-all WGS comparisons using Sourmash (v.3.2.3). Pairwise comparisons were visualized as an all-by-all heatmap (28). Pan-genome analysis was done as previously described (19, 21, 22, 29, 30). Core and accessory genes were annotated using Prokka (version v.1.14.6) (31). Pan-genome comparisons (21) identifying gene clusters and the core genes were conducted using Roary (3.12.0) using 95% amino acid sequence identity (32) and visualized using Phandango (33) with the associated metadata. Gene associations, metadata, and phenotypes were conducted using Scoary 1.6.12 (34).

### Genomic assessment of antimicrobial resistance and virulence factor genes

Antimicrobial resistance and virulence factor genes were analyzed in every genome using ABRicate (version 1.0.0) (35). Antimicrobial resistance genes were screened against the Comprehensive Antibiotic Resistance Database (CARD), the Antibiotic Resistance Gene-Annotation (ARG-ANNOT), MEGARes, ResFinder, and the NCBI Antimicrobial Resistance Gene Finder Plus (AMRFinderPlus). The AMR genes were classified according to the drug class using the CARD database (36). Virulence factor genes were screened against and classified following the VFDB (37). Both AMR and VF determinants were retained for analysis if they fit the minimum criteria of 90% identity and coverage.

### Genotype-phenotype correlation (GPC) for antimicrobial resistance

GPC analysis was performed using broth microdilution antimicrobial susceptibility test results previously published by our research group (38) in combination with the WGS-informed AMR analysis. Antimicrobial susceptibility testing was performed using a microbroth dilution method following the Clinical and Laboratory Standards Institute (CLSI) guidelines (39) with interpretative criteria based on CLSIVET01 standards. Minimum inhibitory concentrations were conducted using the commercially validated panel Sensititre VetBovine BOPO7F plates (Sensititre Susceptibility Plates, Thermo Fisher Scientific, West Sussex, UK), following the manufacturer’s instructions. False-positive, false-negative, sensitivity, specificity, and accuracy of the GPC were calculated as previously described (29, 40). To test the accuracy of WGS-informed AMR analysis identifying MDR isolates, isolates identified as carrying antimicrobial resistance genes belonging to three or more different antimicrobial drug classes were grouped as multidrug resistant, extrapolating from the standard microbiological definition of MDR isolates (41).

### Statistical analysis

Data analysis was conducted using RStudio (version 2024.9.0.375). Descriptive statistics and permutational multivariate analysis of variance were used to examine the distribution of AMR and VF genes in *P. multocida* and *M. haemolytica* between BRD clinical status, age group, and geographic location. *Post hoc* pairwise comparisons with Bonferroni correction were calculated for the categories with significant differences. Proportions of *P. multocida* and *M. haemolytica* genomes with AMR genes were plotted as heatmaps using the pheatmap package. Statistical significance was set for all tests at *P* ≤ 0.05.

## RESULTS

A total of 217 isolates were retrieved from preweaned calves, weaned heifers, and cows were included in the study. *M. haemolytica* (*n* = 68) and *P. multocida* (*n* = 149) genomes were used for population comparisons (Table 1). The *M. haemolytica* genomes ranged from 2.2 to 2.8 Mbp, while the *P. multocida* ranged from 2.0 to 2.7 Mbp (Tables S1 and S2). Additionally, 1,082 *P*. *multocida* and 2,753 *M*. *haemolytica* genomes retrieved from NCBI passed quality thresholds and were used for population analysis (Tables S3 and S4).

**TABLE 1:** Descriptive information for 189 dairy cattle from which *P. multocida* (*n* = 149) and *M. haemolytica* (*n* = 68) isolates were retrieved in California dairy farms^*a*^

		*M. haemolytica*		*P. multocida*	
Variable		BRD cases	BRD control	BRD cases	BRD control
		*n* = 62	*n* = 6	*n* = 131	*n* = 18
Farm	1	14 (82.3%)	3 (17.6%)	53 (84.1%)	10 (15.9%)
	2	16 (84.2%)	3 (15.8%)	35 (83.3%)	7 (16.7%)
	3	32 (100%)	0	43 (97.7%)	1 (2.3%)
Age	Calf	25 (83.3%)	5 (16.7%)	71 (83.5%)	14 (16.5%)
	Heifer	23 (95.8%)	1 (4.2%)	38 (95%)	2 (5%)
	Cow	14 (100%)	0	22 (91.6%)	2 (8.4%)

^*a*^ Percentages represent cumulative percentages of recovering *M. haemolytica* or *P. multocida* by each variable of interest (farm, age).

**Fig 1:**
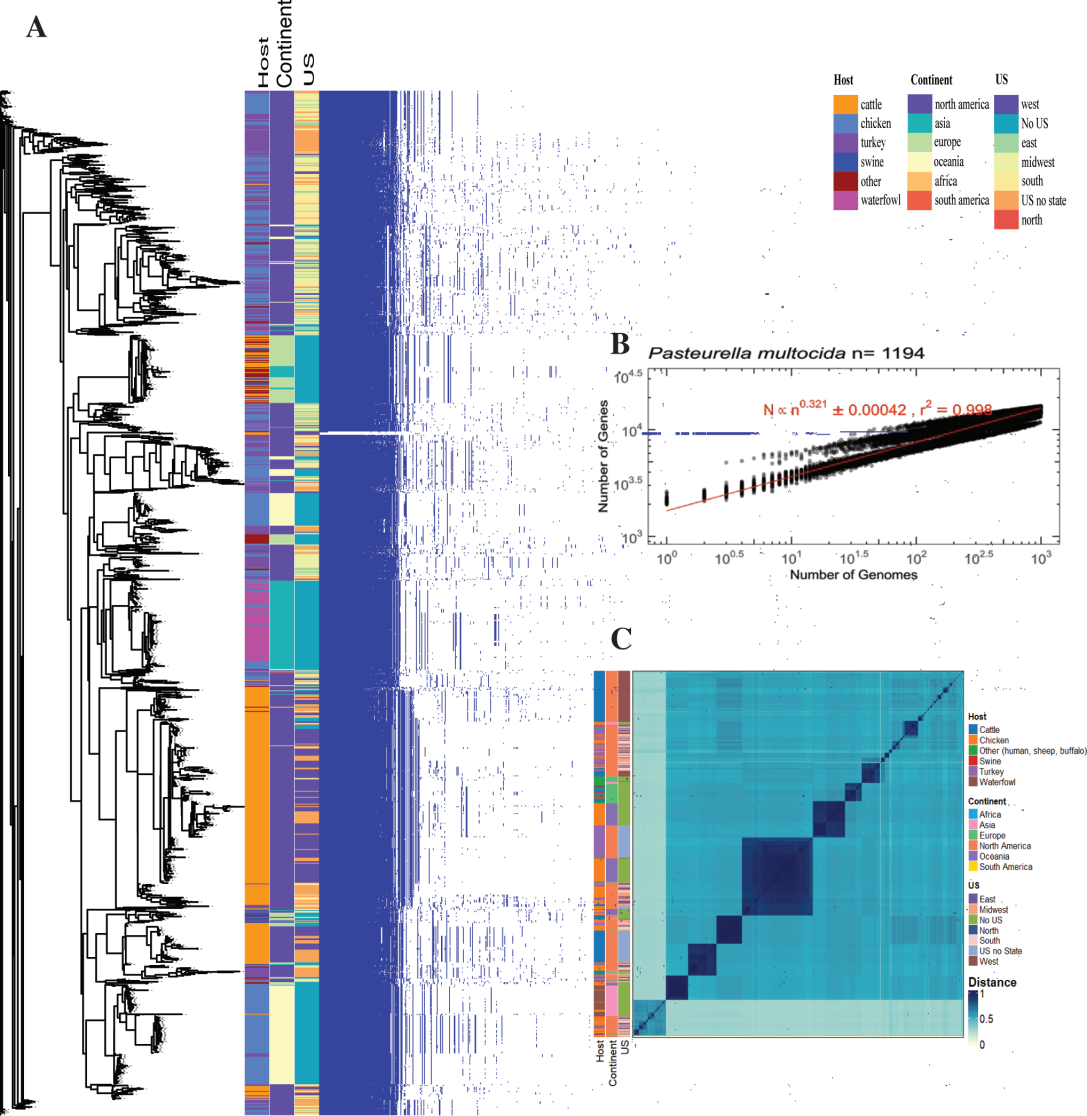
Pan-genome analyses of *P. multocida* isolates. (**A**) Gene presence-absence matrix of the gene distribution in each genome along with the metadata indicating the isolation host (cattle, chicken, swine, turkey, waterfowl, others [human, sheep, buffalo]), the geographic location—continent (Africa, Asia, Europe, North America, Oceania, and South America), the geographic location within USA (not US location, Western, Eastern, Southern**,** Midwest, Northern, no location within US available) of each genome. (**B**) Pan-genome rarefaction curve showing the open pan-genome. (**C**) Whole-genome distance matrix depicting an all-against-all comparison of genome diversity for all isolates associated with the host and geographic location.

**Fig 2:**
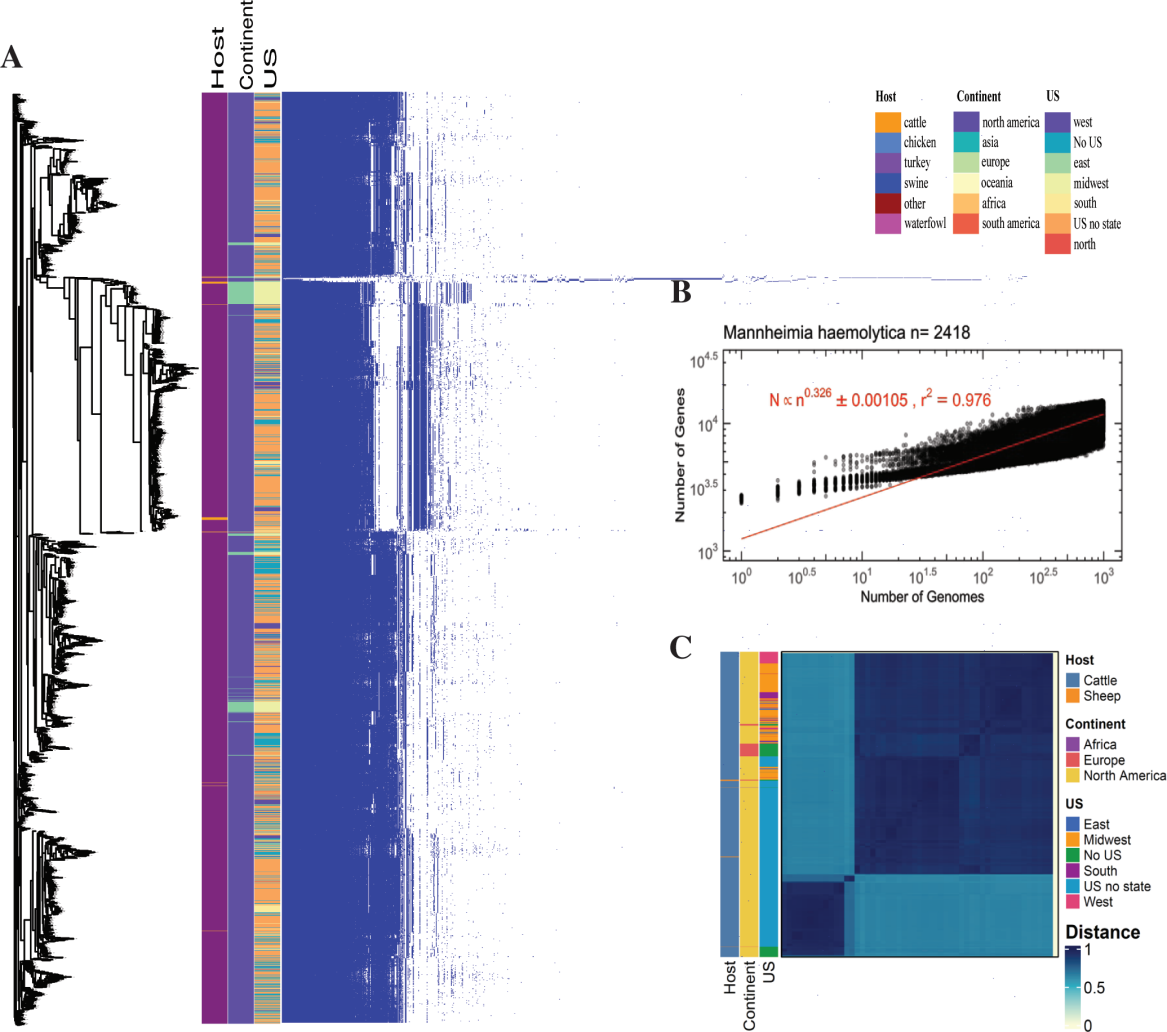
Pan-genome analyses of *M. haemolytica* isolates. (**A**) Gene presence-absence matrix of the gene distribution in each genome along with the metadata indicating the isolation host (cattle, sheep), the geographic location—continent (Africa, Europe, North America, and South America), the geographic location within the USA (not US location, Western, Eastern, Southern**,** Midwest, Northern, no location within the US available) of each genome. (**B**) Pan-genome rarefaction curve showing the open pan-genome. (**C**) Whole-genome distance matrix depicting an all-against-all comparison of genome diversity for all isolates associated with the host and geographic location.

The pan-genome of *P. multocida* was open and was comprised of 16,845 genes, including a core genome of 1,302 genes and soft-core, shell, and cloud genomes with 238, 854, and 14,451 genes, respectively (Fig. 1A). *M. haemolytica*’s pan-genome was open and comprised 16,192 genes, including a core genome of 1,538 genes and soft-core, shell, and cloud genomes with 245, 1,486, and 12,923 genes, respectively (Fig. 2A). The rarefaction curve for gene discovery in *P. multocida* and *M. haemolytica* revealed an exceptionally steep slope that continued without signs of flattening, suggesting an extensive and highly diverse genetic repertoire that remained unexhausted even after analyzing multiple genomes. This persistent steep trajectory indicates that sequencing additional *P. multocida* or *M. haemolytica* genomes is likely to continue revealing novel genes at a substantial rate, highlighting the substantial genomic diversity within both species (42). The mutation frequency in *P. multocida* followed a power law distribution with an exponent of 0.321, while *M. haemolytica* exhibited a slightly higher exponent of 0.326, indicating that both bacteria are rapidly evolving and are prone to mutations, potentially contributing to subtle differences in their evolutionary trajectories and adaptive responses to environmental pressures (Fig. 1B and 2B). In support of this observation, the pan-genome analysis of *P. multocida* and *M. haemolytica* showed that both bacteria are highly diverse, and genomic analyses at the species level did not provide enough resolution to represent the extremely diverse nature of these microorganisms within the species.

Analysis of the population genotype using all publicly available genomes revealed no relationship with BRD status (BRD-positive or healthy control), age (calf, weaned heifer, or cow), and farm (1, 2, or 3), confirming that the population diversity is too high for specific genetic characteristics to be associated with animal traits. All-by-all comparisons revealed no association with geographic location or host (Fig. 1C and 2C). Only when genomes from this study were included was a genotypic relationship with the disease status in either *P. multocida* or *M. haemolytica* found. Three distinct clusters associated with location (farm 2 and 3) and isolates retrieved from heifers were identified for *M. haemolytica* based on the genotype, while multiple clusters related to age (preweaned calves, heifers, and cows) were identified for *P. multocida* (Fig. S1).

**Fig 3:**
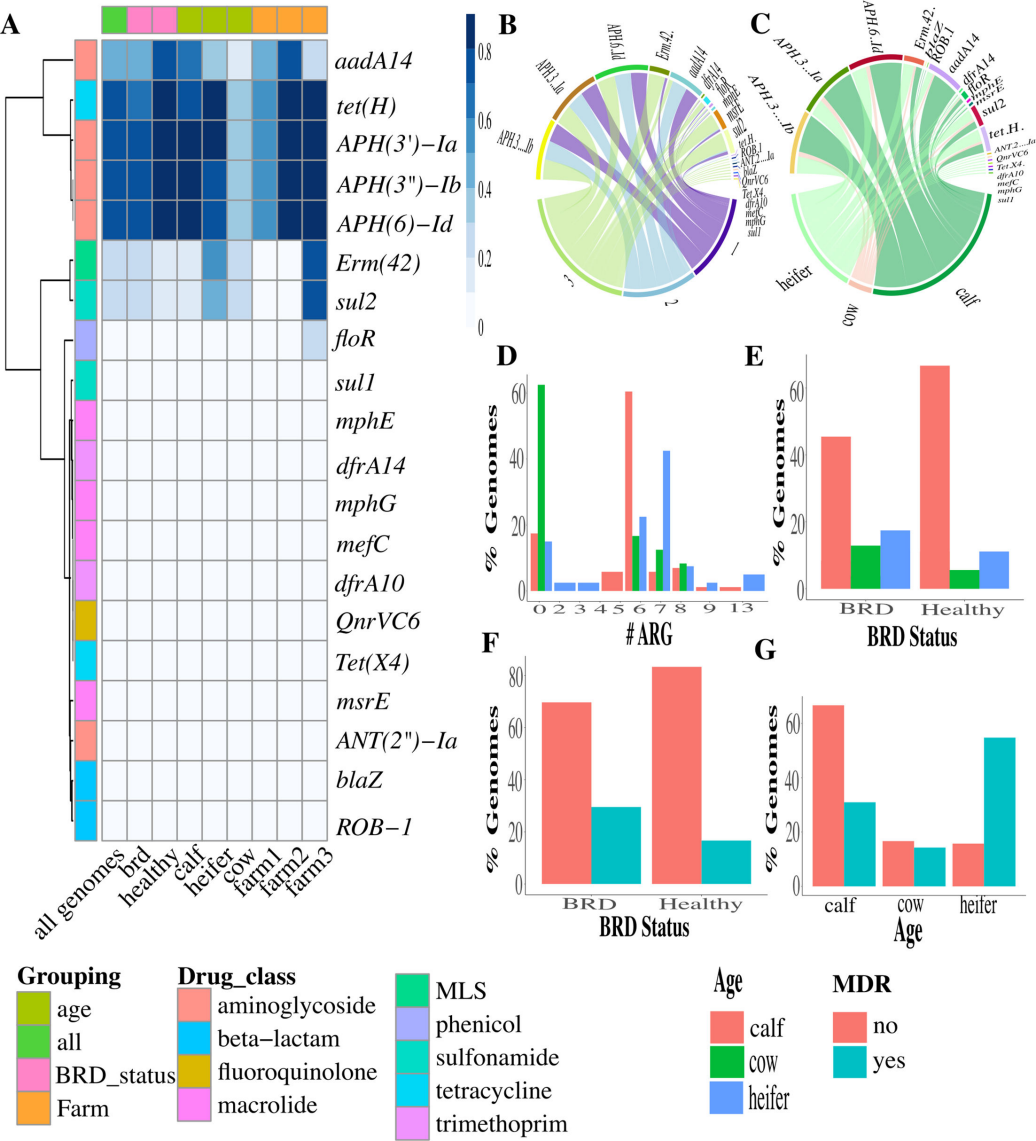
Antimicrobial resistance gene abundance (ARG) as per drug class and age group of *P. multocida* genomes (*n* = 149). (**A**) Heat map of ARG prevalence among isolates. (**B**) Circos plot of association of ARG per farm. (**C**) Circos plot of association of ARG per age group. (**D**) Bar plot of prevalence of genomes with ARG by number of determinants per age group. (**E**) Bar plot of prevalence of genomes with ARG by the number of determinants per health status group. (**F**) Bar plot of prevalence of MDR genomes per health status group. (**G**) Bar plot of prevalence of MDR genomes per age group.

**Fig 4:**
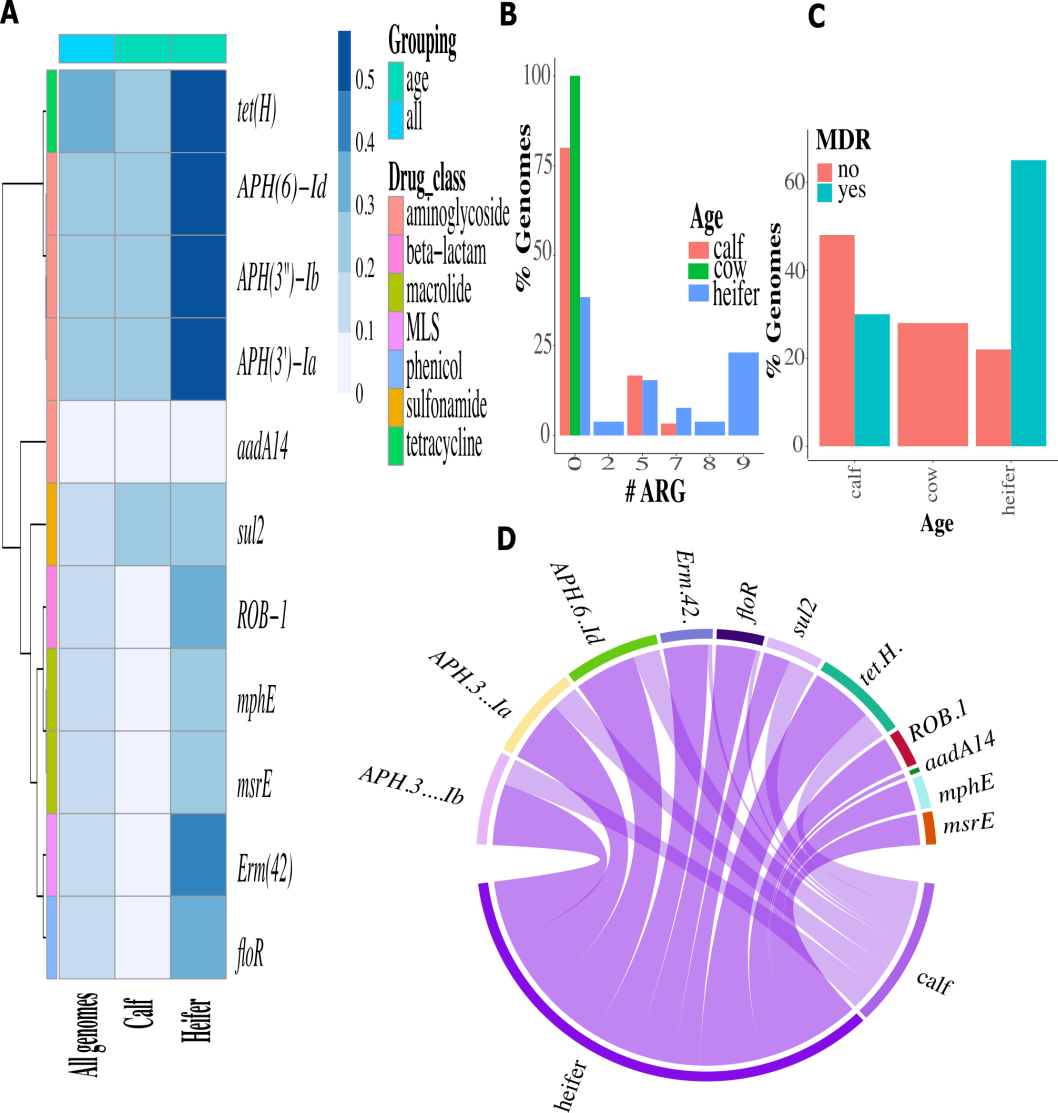
Antimicrobial resistance gene abundance as per drug class and age group of *M. haemolytica* genomes (*n* = 68). (**A**) Heat map of ARG prevalence among isolates. (**B**) Bar plot of prevalence of genomes with ARG by number of determinants per age group. (**C**) Bar plot of prevalence of MDR genomes per age group. (**D**) Circos plot of association of ARG per age group.

Ninety-nine different antimicrobial resistance genes were identified in *P. multocida* and M. *haemolytica* (Fig. S2; Table S7). When only the isolates obtained from this study were evaluated, 22 different antimicrobial resistance genes were identified in *P. multocida* isolates (Fig. 3; Table S8), while 12 different resistance genes were identified in *M. haemolytica* isolates (Fig. 4; Table S8). The most abundant resistance genes found in *P. multocida* at the drug class level were aminoglycosides (76%) and macrolide-lincosamide-streptogramin (27.5%) for the *P. multocida* genomes, and tetracyclines (39.5%) and aminoglycosides (34.3%) for *M. haemolytica*.

**Fig 5:**
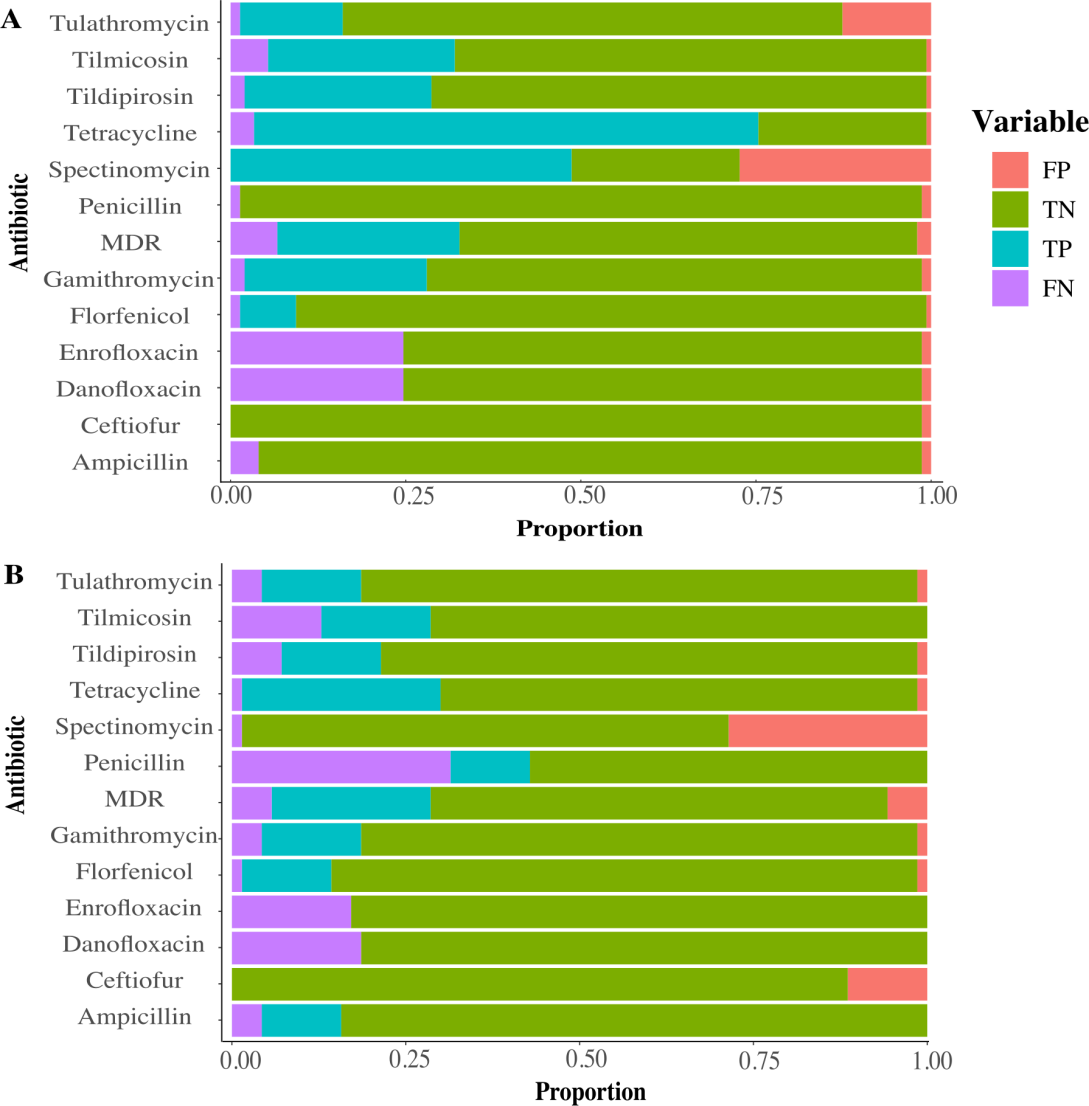
Comparison of phenotypic antimicrobial susceptibility testing and genome-derived resistance prediction for (**A**) *P. multocida* isolates (*n* = 149) and (**B**) *M. haemolytica (n =* 68*).* Color bars correspond to: pink: false-positive (FP), green: true-negative (FN), teal: true-positive (TP), and purple: false-negative (FN).

The correlation between genotype and phenotype revealed inconsistent predictive capabilities using WGS genotype among the antimicrobial agents (Fig. 5). The accuracy of predicting isolate phenotypes ranged widely from 72 to 99% for *P. multocida* and 68 to 96% for *M. haemolytica*. Notably, florfenicol demonstrated the most reliable genotypic-phenotypic correlation for both *M. haemolytica* and *P. multocida*. Tulathromycin had a high prevalence of false positives only for *P. multocida* and ceftiofur for *M. haemolytica* (Tables S5 and S6). Danofloxacin and enrofloxacin had the highest prevalence of false negatives for both *P. multocida and H. haemolytica* (Table S5). Across multiple drugs, the analysis was compromised by a high prevalence of false-positive results (AMR gene identification while the isolate was phenotypically susceptible), significantly reducing the sensitivity of specific antimicrobial genes in identifying drug susceptibility as determined by standard antimicrobial susceptibility testing methods. This result demonstrates that AMR gene presence/absence alone was not an accurate method to predict antimicrobial susceptibility, suggesting that other mechanisms of resistance were possible.

**Fig 6:**
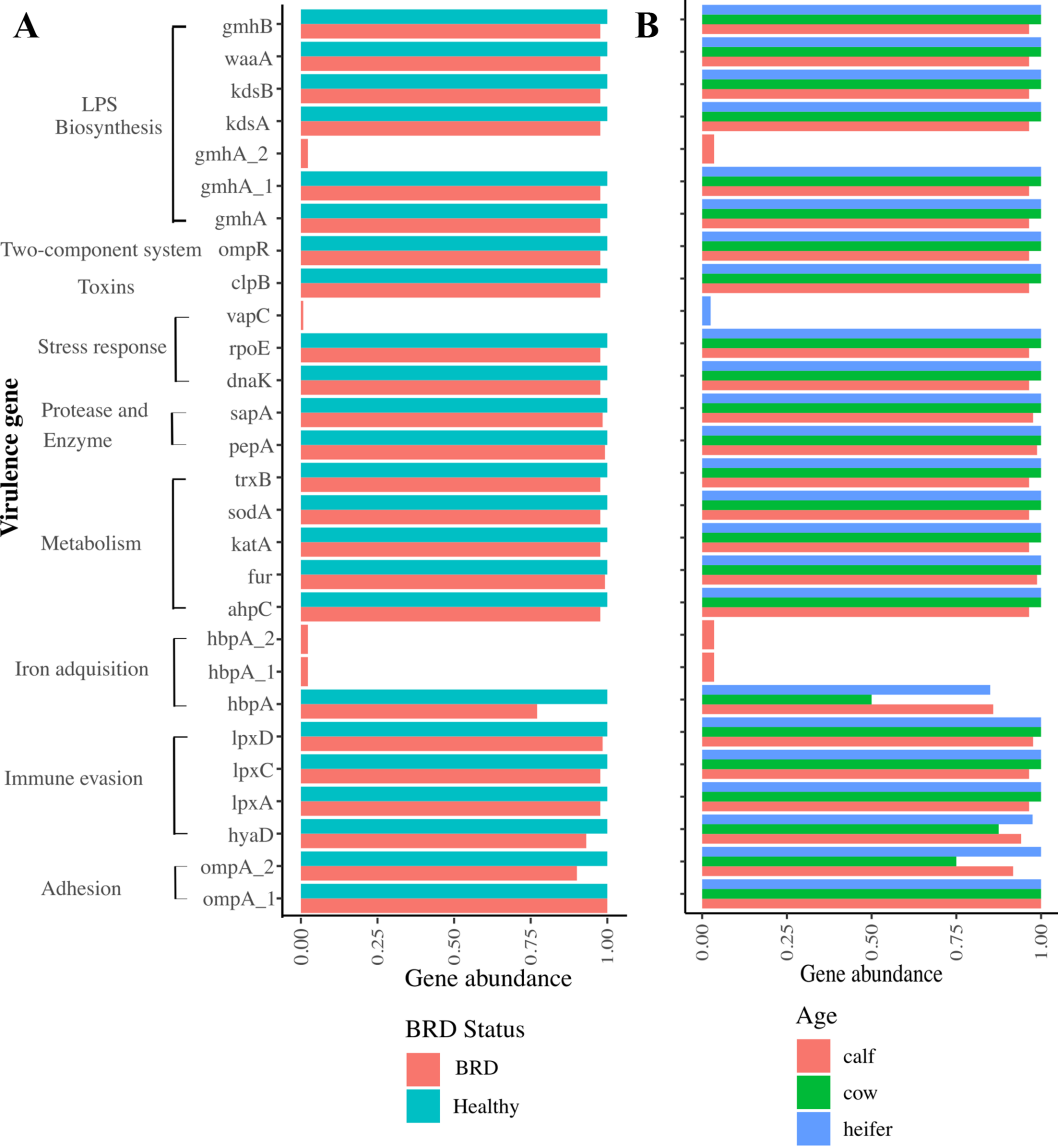
Virulence factor gene abundance for *P. multocida* genomes (*n* = 149) stratified by (**A**) BRD status and (**B**) age group. *LPS: Lipopolysaccharide.*

**Fig 7:**
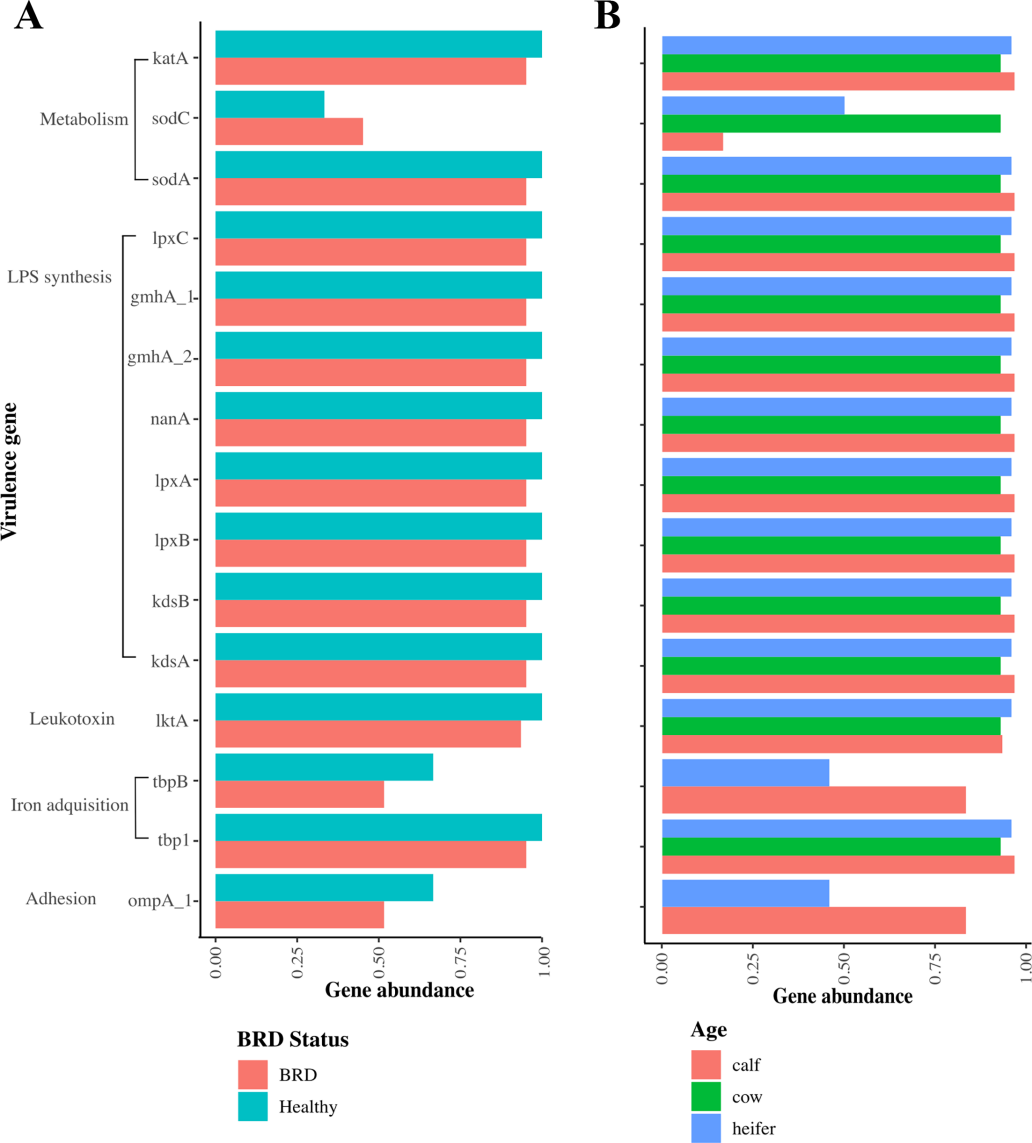
Virulence factor gene abundance for *M. haemolytica* genomes (*n* = 68) stratified by (**A**) BRD status and (**B**) age group.

The number of virulence genes across *P. multocida* or *M. haemolytica* genomes obtained from our study did not significantly differ by BRD status, age, or farm. When genomes were screened against the Virulence Factors Database (VFDB), three virulence genes (*gmhA, lpxC, and rfaD*) were identified in all genomes. Using manual curation, a total of 28 virulence genes belonging to adhesion (*n* = 2), immune evasion (*n* = 4), iron acquisition (*n* = 3), metabolism (*n* = 5), protease and enzyme (*n* = 2), stress response (*n* = 3), toxins (*n* = 1), two-component system (*n* = 1), and Lipopolysaccharide (LPS) biosynthesis (*n* = 7) were detected across the *P. multocida* genomes (Fig. 6). For *M. haemolytica*, 15 virulence genes belonging to adhesion (*n* = 1), iron acquisition (*n* = 2), leukotoxin (*n* = 1), LPS biosynthesis (*n* = 8), and metabolism (*n* = 3) were detected across the genomes (Fig. 7).

## DISCUSSION

BRD is a significant disease in dairy cattle that is persistent and is a substantial cause for the use of antibiotics. Two bacteria, *P. multocida* and *M. haemolytica*, are associated with the development of BRD. Because these organisms are common commensals in the respiratory tract, it has been difficult to diagnose and treat this disease. To examine the cause of this disease, we conducted a population genomic comparison of *P. multocida* and *M. haemolytica* isolates from both BRD-affected and healthy cattle across three age groups, with a focused set of isolates from this study, along with an analysis that used all available genomes for these two organisms, with the hypothesis that identifying AMR genes and VFs will be associated with disease occurrence and severity.

To our knowledge, this is the first report using a case-control study design, including different age groups, and combined with WGS to define the AMR and VF genes in bacteria associated with BRD in dairy cattle. Previous studies have typically used a single age group or a cross-sectional study design (10, 43–47). Cross-sectional study designs described the AMR and VF genes in bacteria isolated from BRD-affected animals; however, it is unclear how the relative abundance of these genes differs between healthy and diseased animals and between age groups, which is crucial for understanding BRD epidemiology and informing treatment strategies.

The gene content from the pan-genome analysis for both *M. haemolytica* and *P. multocida* did not differentiate between cases and controls, isolation hosts, age, or location. Genotypic analysis found that the genomes were open and highly mutable (power law calculations), indicating the population is extremely diverse and easily mutable. The lack of differentiation among bacteria regardless of the isolation source was likely related to the high genomic diversity of the bacteria. BRD-associated traits were not associated in gene presence-absence patterns, suggesting that mutational changes or multigenic association is possible, in addition to what is established with disease development and host factors and microbial-host interactions than bacterial genetic content. Both bacteria are commensal microorganisms in the upper respiratory tract of healthy cattle but can rapidly transition to opportunistic pathogens when host defenses are compromised by stress, viral infections, or environmental factors, descending to the lower respiratory tract where they proliferate and cause BRD (48–50). Further research should explore case-control studies along with gene expression, microbiome composition, and host-pathogen interactions to better understand BRD pathogenesis.

This study found no significant association between BRD cases or healthy controls, isolation host, or geographic location for antimicrobial resistance gene occurrence in the microbial population. However, the prevalence of genomes containing AMR genes differed when stratified by age for both *P. multocida* and *M. haemolytica*, with the highest prevalence among preweaned calves when compared to weaned heifers and cows. Similar findings have been reported where the preweaned calves showed the highest prevalence of AMR genes, which could be explained by the increased exposure and use of antimicrobials in young livestock to prevent and treat infectious diseases, as well as exposure to medicated milk replacer or waste milk containing antimicrobial residues (51–53).

This study found that the prevalence of genomes with an MDR profile was higher in weaned heifers for both *P. multocida* and *M. haemolytica* compared to preweaned calves and cows. Previous studies have found that AMR in cattle often decreases with age (51), but the AMR profile observed in our study indicated an increase in AMR from calves to heifers (54). Multiple reasons could have contributed to this higher MDR prevalence, such as previous antimicrobial exposure or housing characteristics. Further longitudinal studies evaluating the differences in AMR by age groups are needed to understand better the causes and associated risk factors of MDR bacteria emergence, persistence, and dissemination in dairy cattle.

Genes encoding resistance to multiple critically important antimicrobial classes, such as beta-lactams, macrolides, and tetracyclines, were found in this study. The emergence and spread of antimicrobial resistance in livestock production systems is a major global animal and public health threat (55–57). The widespread use of antimicrobials in animal production is one of many factors contributing to the selection and dissemination of antimicrobial-resistant bacteria (57). Additionally, from a livestock medicine perspective, the increase in antimicrobial resistance in clinically relevant bacteria poses a threat to effectively treating infectious diseases such as BRD. The widespread detection of antimicrobial resistance genes across multiple age groups in our study underlines the critical importance of implementing comprehensive surveillance programs to monitor antimicrobial resistance in livestock production systems.

The phenotype-genotype correlation showed that WGS was moderately successful at predicting antimicrobial resistance overall using gene presence-absence, with accuracy varying across different types of antimicrobial drugs tested. These results agree with other studies in which the concordance varied significantly based on the bacteria and antimicrobial class being compared (10, 45, 58). The most common discrepancy between phenotype and genotype antimicrobial susceptibility was observed for the fluoroquinolone group, which was led by a higher prevalence of false-negative results than the other antimicrobial classes. Although isolates were phenotypically resistant, no antimicrobial resistance genes coding for this drug class were identified. For both *M. haemolytica* and *P. multocida*, the only gene identified through WGS was *QnrVC,* which is commonly plasmid-mediated and confers quinolone resistance gene that belongs to the *qnr* gene family (59, 60). These genes encode proteins that protect DNA gyrase and topoisomerase IV from quinolone antibiotics, contributing to resistance against fluoroquinolones. However, there is substantial evidence for the role of chromosomal mutations in fluoroquinolone resistance in *Pasteurellaceae*, especially mutations on *gyrA* and *parC*, instead of plasmid-mediated quinolone resistance genes such as *QnrVC*.(61–64) Similar findings were reported in a California study comparing 326 bacterial respiratory isolates from weaned dairy heifers, for which fluoroquinolone agreement was poor due to the lack of identification of fluoroquinolone AMR genes in any of the isolates displaying phenotypic resistance (10). This suggests that our current understanding of the genetic mechanisms underlying resistance to these antimicrobial classes remains incomplete and that further research is needed to elucidate the full repertoire of resistance determinants in these and other bacterial pathogens (65–67).

Additionally, databases used for AMR are not comprehensive enough to capture all mechanisms of resistance, especially for certain understudied bacteria such as *P. multocida* and *M. haemolytica* (58, 66, 67). This work used five different databases to find only two genes that were not contained in one database. However, the lack of total genomes in this organism and the high genome flexibility add to the complication of database completeness. Additional work is needed to curate databases for these genetically diverse organisms to increase discovery of genes linked to AMR.

This work revealed no significant differences in the prevalence of virulence genes between *P. multocida* and *M. haemolytica* isolates. These results align with prior research that examined *P. multocida* isolates from both healthy (*n* = 14) and BRD-affected (*n* = 14) feedlot cattle (45). That study identified 27 distinct virulence genes, with 21 of these genes presented across all genomes, and similarly found no correlation between specific virulence genes and animal health status. Both *P. multocida* and *M. haemolytica*’s high genomic diversity make it challenging to establish clear associations between genetic traits and health status based solely on gene presence or absence using WGS. Incomplete annotation of both genomes may significantly hinder genomic comparisons, as many AMR or VF genes may not have been correctly identified, or they have not been elucidated to date. Further research exploring host-microbe interactions is needed to better understand disease pathogenesis and identify genomic variations that distinguish commensal from pathogenic bacteria involved in BRD, as well as efforts to diversify sampling, experimental validation of hypothetical proteins, advanced computational methods leveraging machine learning and comparative genomics, and improved database interoperability to enhance our understanding of these economically significant livestock pathogens.

### Conclusion

A BRD-specific genotype was not found to be associated with *M. haemolytica* or *P. multocida* in this study. In contrast, a high diversity among isolates, regardless of disease status, was observed. Twenty-two AMR genes for 10 antimicrobial classes were identified in *P. multocida* isolates, while 12 AMR genes for seven antimicrobial classes were identified in *M. haemolytica* isolates. VF genes were identified in *P. multocida* (28 genes) and *M. haemolytica* (15 genes). The high genetic diversity observed in both bacterial species prevented the identification of specific genetic markers associated with disease status. The ability of WGS to predict phenotypic AMR showed variable accuracy across different antimicrobials, achieving moderate levels of agreement overall. Findings from this study demonstrate that the identification of genomic markers based on gene presence-absence lacks discriminatory power in identifying unique genotypes associated with disease status and indicates other factors may have a stronger association with BRD occurrence. Further research that investigates the respiratory tract microbiome in cattle with and without bovine respiratory disease, as well as the role of the host immune response and host-microbe interaction in disease development and across different age groups, may help better understand BRD pathophysiology and tailor interventions for disease diagnosis and successful treatment.

## Data Availability

All raw genome sequences generated in this study are available at the 100K Pathogen Genome Project BioProject (NCBI PRJNA186441) under BioProject accession number PRJNA1267165.

## SUPPLEMENTAL MATERIAL

Supplemental figures spectrum.01200-25-s0001.docx Figures S1 and S2.

Supplemental tables spectrum.01200-25-s0002.xlsx Tables S1 to S8.

ASM does not own the copyrights to Supplemental Material that may be linked to, or accessed through, an article. The authors have granted ASM a non-exclusive, world-wide license to publish the Supplemental Material files. Please contact the corresponding author directly for reuse.
