# Supplementary material for: Comparison of virulence and resistance genes in Mannheimia haemolytica and Pasteurella multocida from dairy cattle with and without bovine respiratory disease
Source: Microbiol Spectr. 2025 Jun 16;13(8):e01200-25. doi: 10.1128/spectrum.01200-25 (PMC12323314; doi:10.1128/spectrum.01200-25)
Supplement: Supplemental figures — Figures S1 and S2. [file spectrum.01200-25-s0001.docx]

**Comparison of virulence and resistance genes in *Mannheimia haemolytica* and *Pasteurella multocida* from dairy cattle with and without bovine respiratory disease**

Adriana Garzon^1^, Craig Miramontes^1^, Bart C Weimer^1,2*^, Rodrigo Profeta^1,2^, Alejandro Hoyos-Jaramillo^1^, Heather M Fritz^3^, Richard V Pereira^1*^

^1^Department of Population Health and Reproduction, School of Veterinary Medicine, University of California, Davis, CA, USA

^2^100K Pathogen Genome Project, School of Veterinary Medicine, University of California, Davis, CA, USA

^3^California Animal Health and Food Safety Lab, University of California, Davis, CA, USA

^*^**Corresponding authors:** Drs. Richard V. Pereira ([rvpereira@ucdavis.edu](mailto:rvpereira@ucdavis.edu)) and Bart C. Weimer ([bcweimer@ucdavis.edu](mailto:bcweimer@ucdavis.edu))

**Supplemental Figure 1.** Whole-genome distance matrix depicting an all-against-all comparison of genome diversity for isolates associated with age, farm and status for **A.** *P. multocida* and **B.** *Mannheimia haemolytica*


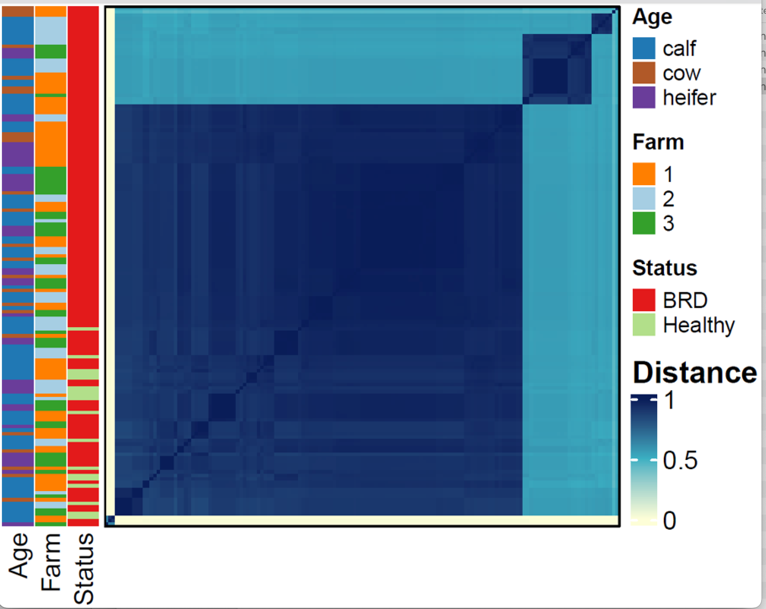


**A**

**B**


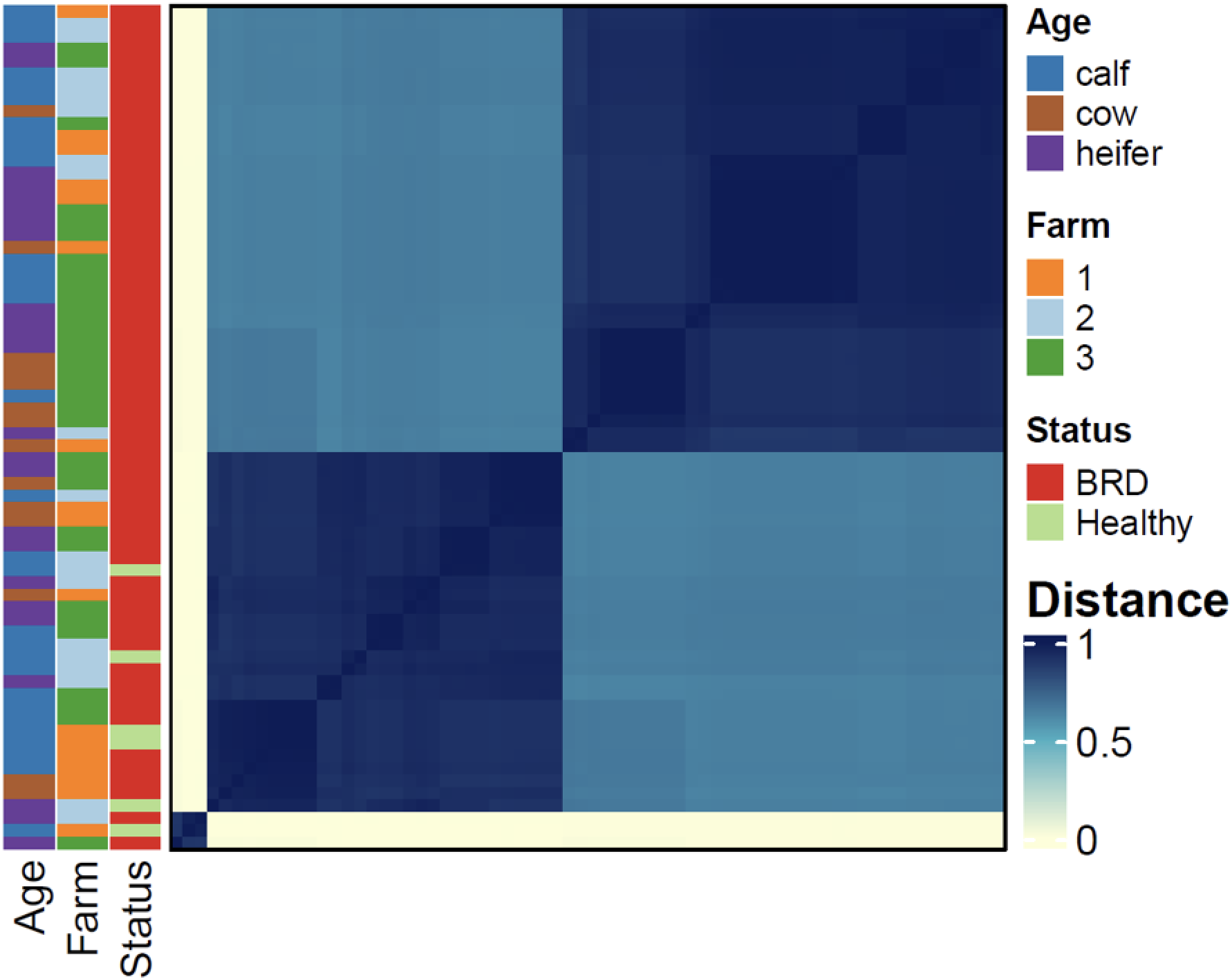


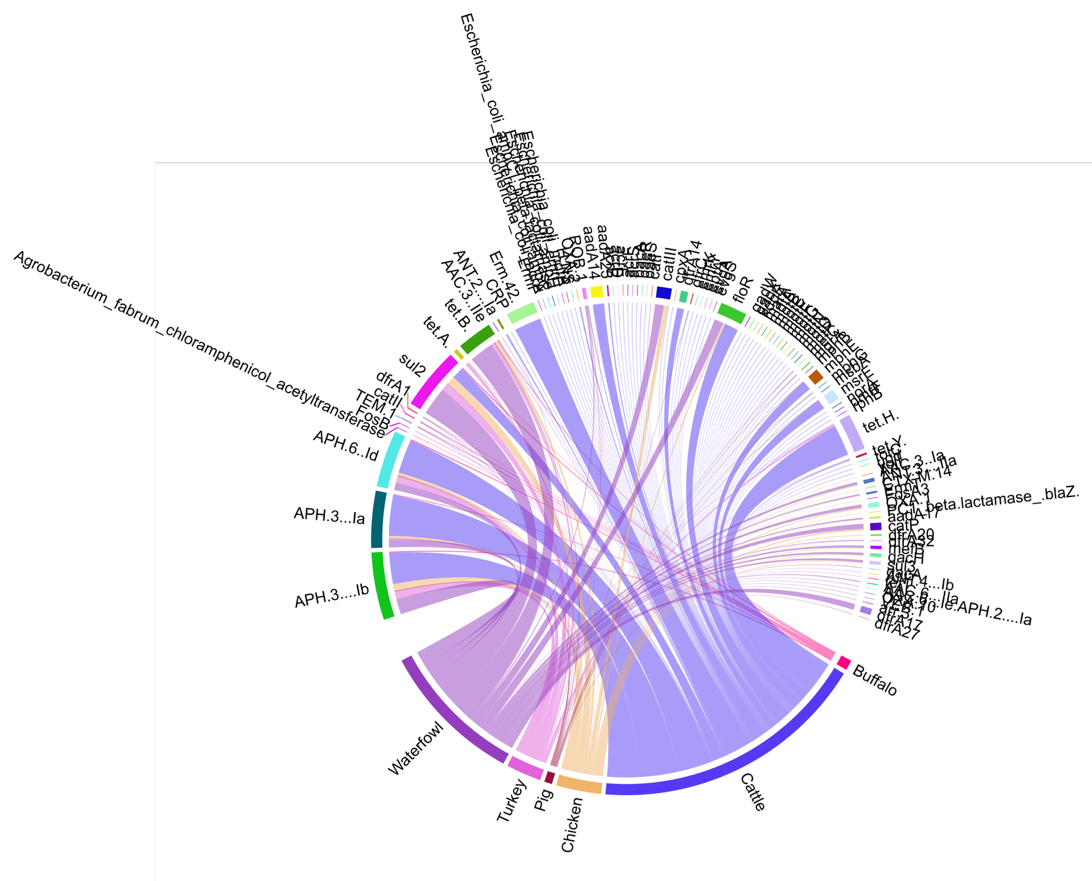
**Supplemental Figure 2.** Circos plot of association of antimicrobial resistance per host and antimicrobial drug class (A, B), and host and antimicrobial resistance genes (C, D) in *P. multocida* (n=1,082) and *M. haemolytica* (n=2,574).


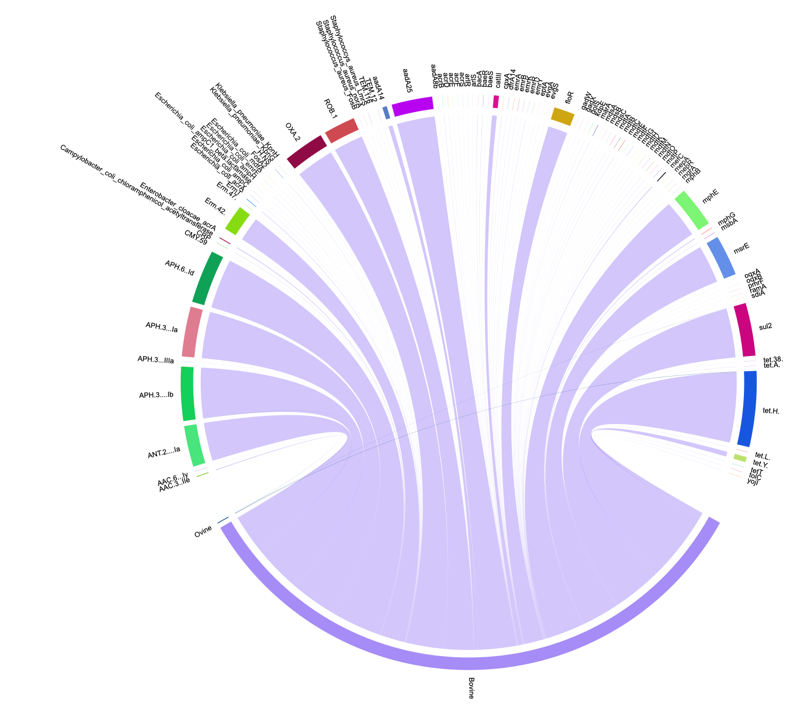


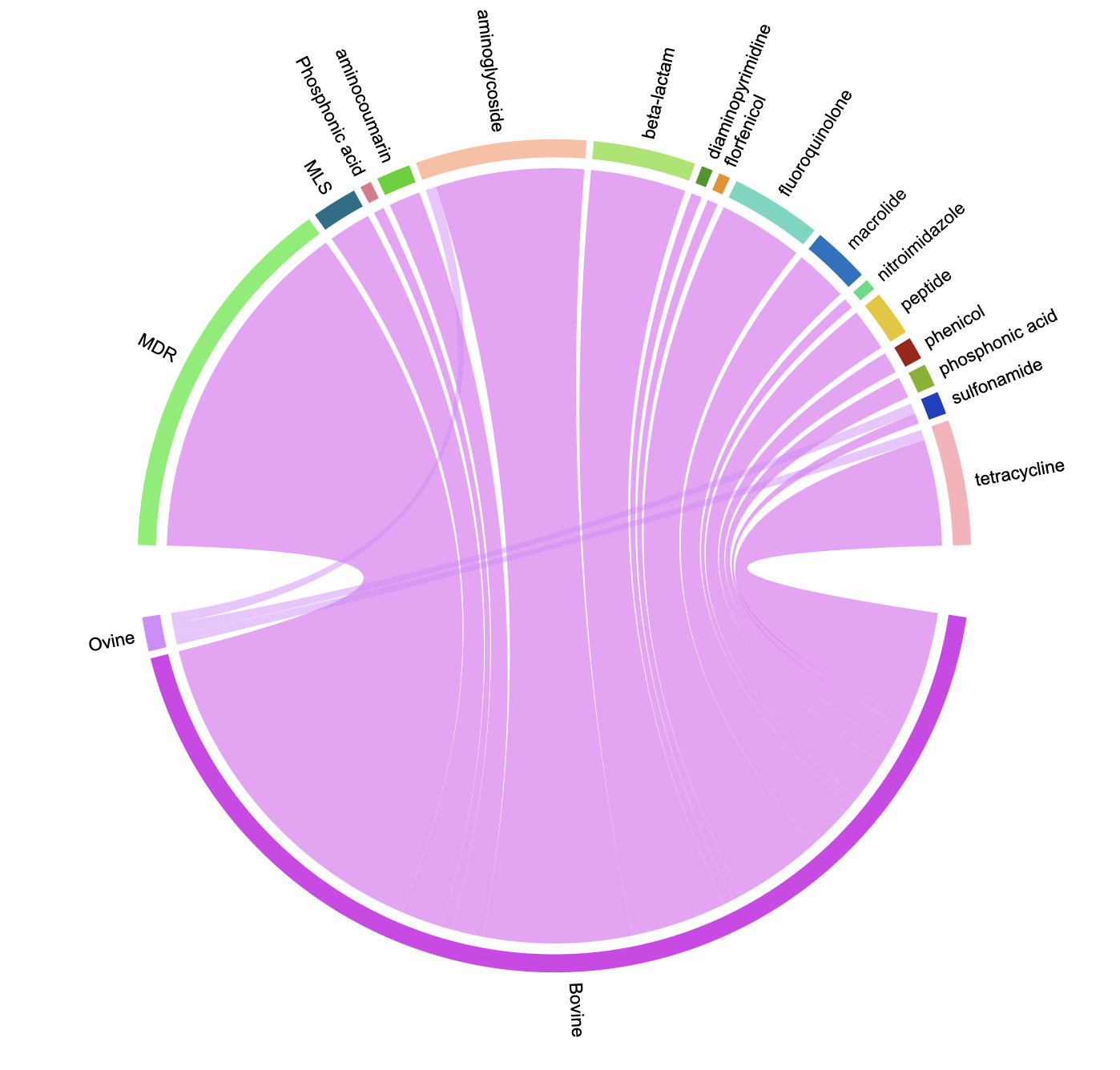


**B**

**D**

**A**


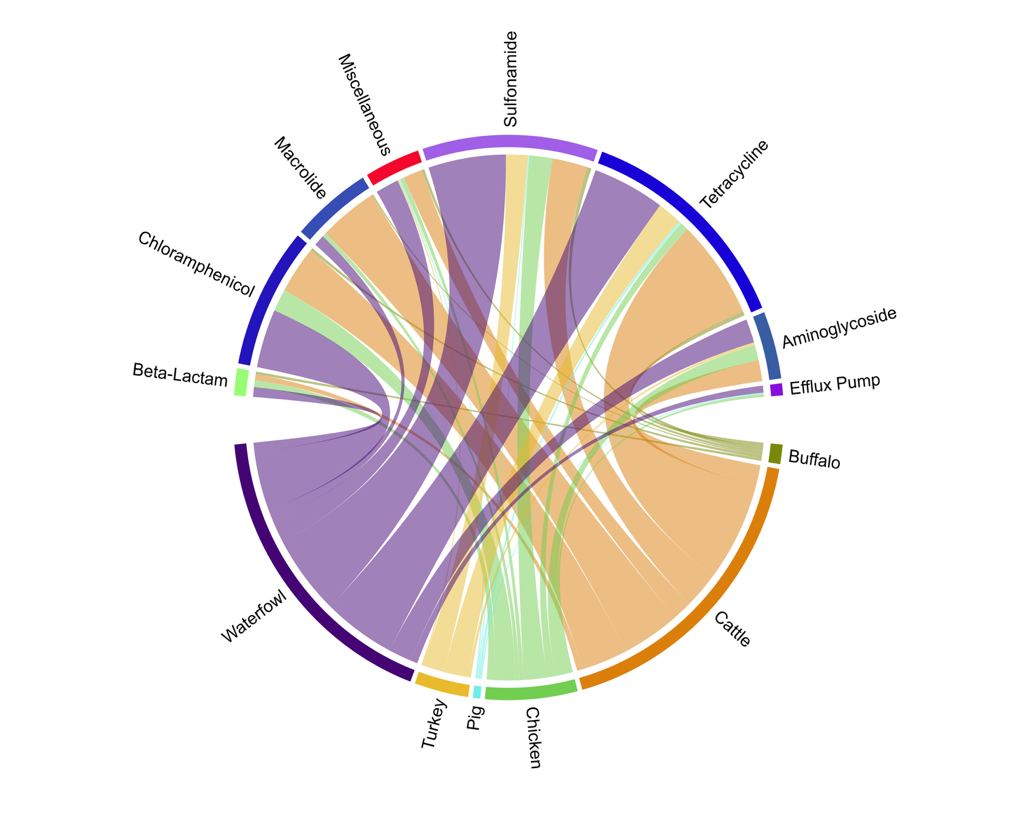


*M. haemolytica*

**C**

*P. multocida*
